# Six-month periodic fasting does not affect somatosensory nerve function in type 2 diabetes patients

**DOI:** 10.3389/fendo.2023.1143799

**Published:** 2023-05-12

**Authors:** Zoltan Kender, Ekaterina von Rauchhaupt, Daniel Schwarz, Dimitrios Tsilingiris, Lukas Schimpfle, Hannelore Bartl, Valter D. Longo, Martin Bendszus, Stefan Kopf, Stephan Herzig, Sabine Heiland, Julia Szendroedi, Alba Sulaj

**Affiliations:** ^1^ Clinic for Endocrinology, Diabetology, Metabolic Diseases and Clinical Chemistry (Internal Medicine 1), Heidelberg University Hospital, Heidelberg, Germany; ^2^ German Center of Diabetes Research (DZD), Neuherberg, Germany; ^3^ Department of Neuroradiology, Heidelberg University Hospital, Heidelberg, Germany; ^4^ Longevity Institute, School of Gerontology, and Department of Biological Sciences, University of Southern California, Los Angeles, CA, United States; ^5^ FIRC Institute of Molecular Oncology, Italian Foundation for Cancer Research Institute of Molecular Oncology, Milan, Italy; ^6^ Institute for Diabetes and Cancer, Helmholtz Center Munich, Neuherberg, Germany; ^7^ Joint Heidelberg-IDC Translational Diabetes Program, Internal Medicine 1, Heidelberg University Hospital, Heidelberg, Germany; ^8^ Chair Molecular Metabolic Control, Technical University Munich, Munich, Germany; ^9^ Joint Heidelberg-IDC Translational Diabetes Program, Helmholtz Center Munich, Neuherberg, Germany

**Keywords:** type 2 diabetes mellitus, diabetic sensorimotor polyneuropathy, periodic fasting, nerve conduction velocity, hypoalgesia

## Abstract

**Background and aim:**

Current strategies for preventing diabetic sensorimotor polyneuropathy (DSPN) are limited mainly to glucose control but rapid decrease of glycemia can lead to acute onset or worsening of DSPN. The aim of this study was to examine the effects of periodic fasting on somatosensory nerve function in patients with type 2 diabetes (T2D).

**Study design and methods:**

Somatosensory nerve function was assessed in thirty-one patients with T2D (HbA1c 7.8 ± 1.3% [61.4 ± 14.3 mmol/mol]) before and after a six-month fasting-mimicking diet (FMD; n=14) or a control Mediterranean diet (M-diet; n=17). Neuropathy disability score (NDS), neuropathy symptoms score (NSS), nerve conduction velocity and quantitative sensory testing (QST) were analyzed. 6 participants of the M-Diet group and 7 of the FMD group underwent diffusion-weighted high-resolution magnetic resonance neurography (MRN) of the right leg before and after the diet intervention.

**Results:**

Clinical neuropathy scores did not differ between study groups at baseline (64% in the M-Diet group and 47% in the FMD group had DSPN) and no change was found after intervention. The differences in sensory NCV and sensory nerve action potential (SNAP) of sural nerve were comparable between study groups. Motor NCV of tibial nerve decreased by 12% in the M-Diet group (P=0.04), but did not change in the FMD group (P=0.39). Compound motor action potential (CMAP) of tibial nerve did not change in M-Diet group (P=0.8) and increased in the FMD group by 18% (P=0.02). Motor NCV and CMAP of peroneal nerve remained unchanged in both groups. In QST M-diet-group showed a decrease by 45% in heat pain threshold (P=0.02), FMD group showed no change (P=0.50). Changes in thermal detection, mechanical detection and mechanical pain did not differ between groups. MRN analysis showed stable fascicular nerve lesions irrespective of the degree of structural pathology. Fractional anisotropy and T2-time did not change in both study groups, while a correlation with the clinical degree of DSPN could be confirmed for both.

**Conclusions:**

Our study shows that six-month periodic fasting was safe in preserving nerve function and had no detrimental effects on somatosensory nerve function in T2D patients.

**Clinical trial registration:**

https://drks.de/search/en/trial/DRKS00014287, identifier DRKS00014287.

## 1 Introduction

One in ten adults is living with diabetes and the incidence is rising (1, 2). Diabetic sensorimotor polyneuropathy (DSPN) is the most common complication of diabetes mellitus (3) and the prevalence increases with diabetes duration reaching up to 50% at 10 years after diagnosis (4, 5). Hyperglycemia and dyslipidemia lead to alterations in glucose and lipid metabolism that deteriorate neuronal function and contribute to DSPN through production and accumulation of reactive oxygen species (ROS) and advanced glycation end-products (AGEs) (6). Current treatment strategies focus on glycemic control and target mainly the positive symptoms of DSPN such as pain and paresthesia (7, 8) leaving individuals with negative symptoms of DSPN with limited therapeutic options.

Glycemic control has been shown to be effective in delaying the onset and progression of DSPN in individuals with type 1 diabetes but no similar effects could be confirmed in those with type 2 diabetes (T2D) (5, 9). Studies have demonstrated an improvement in neuropathy outcomes after lifestyle modifications in patients with neuropathy including long-term aerobic training (10) and intensive life-style interventions (11). Although these studies investigated the course of DSPN during long-term interventions (4 and 9-11 years respectively), study participants at initial presentation had either no signs and symptoms of DSPN (10) or only some mild evidence of DSPN (11), increasing the likelihood of the intervention to prevent the progress of DSPN. Moreover, DSPN characterization was based only on clinical neuropathy scores and nerve conduction velocity analysis, whereas no data on quantitative sensory testing or MRI nerve lesions were collected. Diet intervention that induce ketogenesis and lipolysis, either through fat-favorable composition of energy sources, or through fasting and caloric intake reduction, have beneficial disease-modifying effects on different neurological disorders including epilepsy (12), multiple sclerosis (13) and central spine injury in humans (14) and in animal models (15) mediated through alterations in extracellular signal-regulated kinases (ERK)- and mammalian target of rapamycin (mTOR)-signaling (16) and in opioid-receptor expression (17). On the other hand, diet intervention in diabetes patients should be considered carefully, since rapid decrease of glycemia increases the risk of treatment-induced neuropathy in diabetes (TIND) (18).

We have recently shown that six-month periodic fasting can improve microalbuminuria and metabolic control in patients with T2D and diabetic nephropathy (19). The aim of the current study was to investigate possible protective effects of periodic fasting on DSPN by assessing clinical, electrophysiological, and magnetic resonance neurography parameters in individuals with T2D.

## 2 Methods

### 2.1 Study design and study population

Individuals of this study were part of a randomized controlled study at the Clinic of Endocrinology, Diabetology, Metabolic Diseases and Clinical Chemistry at the University of Heidelberg in Germany. The study protocol was approved by the ethics committee of the University of Heidelberg (Ethic-Nr. S-682/2016) in compliance with national guidelines and the declaration of Helsinki. This study was registered at the German Clinical Trials Register (Deutsches Register Klinischer Studien DRKS; DRKS-ID: DRKS00014287).

Inclusion criteria were age between 50 and 75 years, T2D with a disease duration of minimum 1 year, estimated glomerular filtration rate (eGFR) > 30 ml/min/173m^3^ and body mass index between 23 to 40 kg/m^2^. Individuals with known neurological diseases or neuropathy-associated risk factors such as entrapment syndromes, multiple sclerosis, lumbar surgery or disc extrusion, Parkinson´s disease, alcohol abuse, hypovitaminosis, malignant or infectious diseases were excluded. Full list of exclusion criteria has been previously published (19).

Eligible study participants were randomly assigned to receive either a fasting-mimicking diet (FMD) or a Mediterranean diet (M-Diet) for 5 consecutive days each month. Participants in the FMD group received a plant-based diet that mimics fasting-like effects on glucose and ketone bodies as previously reported (19). Participants of the M-Diet group were instructed to follow for five days an isocaloric Mediterranean diet. After 5 days of diet intervention the participants of both study groups returned to their normal diet until the next diet cycle was initiated about 3 weeks later. The diet cycles were repeated every month and for six months in total.

### 2.2 Blood chemistry

Blood samples were drawn in fasting state and immediately processed in the Central Laboratory of the University Hospital of Heidelberg under standardized conditions. Beta-hydroxybutyrate was measured at each visit in venous blood (StatStrip^®^ Glucose/Ketone Meter System, Nova^®^ Biomedical).

### 2.3 Neuropathy assessment

Neuropathy assessment was performed at baseline and aftersix months according to the guidelines of the German Society for Diabetology based on the neuropathy symptom score (NSS) and the neuropathy disability score (NDS) (20, 21). Electrophysiological examination of the right leg was performed using a Viking IV electromyography system (Viasys Healthcare, France) on peroneal, tibial and sural nerves. DSPN was determined by a score of ≥3 in the NDS and NSS as described previously (22) (23). Quantitative sensory testing (QST) was performed on the right foot (testing area) and on the right hand (inter-individual control area) to determine the subjective detection thresholds for sensory stimuli and to assess for specific sensitivity of differently myelinated neuronal fibers in the tested regions: thickly myelinated afferents (low-threshold mechanoreceptors for touch and vibration), thinly myelinated afferents (cold fibers and mechano-nociceptors), unmyelinated afferents (warm fibers, heat-sensitive polymodal nociceptors). Electrophysiological examination was performed by one experienced medical technician and QST was performed by two experienced medical technicians throughout the study. All investigators performing QST were trained and certified by the Department of Neurophysiology at the University Hospital of Mannheim. For evaluating small fiber function QST was performed according to the protocol of the “German Neuropathic Pain Research Network” (DFNS) by use of a thermode (TSA-II; Medoc Ltd., Ramat Yishai, Israel) as previously described (21, 24, 25). Complete QST can determine neuropathic deficits and hyperalgesia with the following parameters: cold detection threshold (CDT); warm detection threshold (WDT); thermal sensory limen (TSL); cold pain threshold (CPT); heat pain threshold (HPT); pressure pain threshold (PPT); mechanical pain threshold (MPT); mechanical pain sensitivity (MPS); wind-up ratio (WUR); mechanical detection threshold (MDT); vibration detection threshold (VDT); dynamic mechanical allodynia (DMA); and paradoxical heat sensation (PHS). Results from each evaluated QST parameter were compared to reference values of DFNS. These reference values ± standard deviations (adjusted to age and gender) were used to calculate z-scores for all single tests except PHS and DMA, as described earlier (24). The z-values of the single tests were clustered to create composite z-scores as previously described (26): thermal detection (average values of CDT, WDT, and TSL, demonstrating thermo-receptive Aδ- and C-fiber function), thermal pain (average values of CPT and HPT, representing nociceptive C-fiber function), mechanical detection (average values of VDT and MDT, demonstrating tactile Aβ fiber function), and mechanical pain (average values of MPT, MPS, and PPT, representing nociceptive Aδ and C-fiber function).

### 2.4 Magnetic resonance neurography

To detect peripheral nerve lesions and the microstructure integrity of the sciatic nerve, thirteen participants, of whom 6 were allocated to the M-Diet group and 7 to the FMD group, underwent high-resolution T2-weighted anatomical magnetic resonance neurography (MRN) with fat spectral saturation, T2-relaxometry and diffusion-tensor-imaging (DTI) of the right thigh in a 3.0 Tesla MRI scanner (Magnetom Prisma-Fit; Siemens Healthineers, Germany; 15-channel transmit–receive knee coil) at baseline and at the end of intervention after six months. Sciatic nerve’s fractional anisotropy (FA), commonly considered the most sensitive marker of nerve fiber integrity, was calculated automatically from the DTI dataset using the built-in algorithm. T2-timewas determined from the T2 relaxometry dataset using a custom-written MatLab routine based on monoexponential fitting procedure (R2020a, MathWorks Inc., Natwick, MA, USA) as reported previously (27).

### 2.5 Power calculation

Based on previous studies (28) and on our clinical experience, we considered a change of the clinical neuropathy scores by at least 50% as clinically relevant. Thus, the actual study required a total sample size of 28 patients to detect a mean difference between groups of 50% decrease of NSS/NDS from baseline, assuming an SD of 45% for both groups with a 2-sample t-test and a 2-sided significance level of α = 5% and a power of at least 80%. Sample size calculation was performed in PASS 16.0.12.

### 2.6 Statistical analysis

Analyses were performed in the modified intention-to-treat subpopulation as a complete case analysis, with available neuropathy assessment data at baseline and after 6 months. To check if the randomization was still valid in the subpopulation, a Mann-Whitney test was applied for comparison of baseline values. Descriptive data are shown as mean ± SD for normally distributed variables, median (interquartile range) for log-normally distributed variables, and frequencies for categorical variables. Distribution was evaluated by the Kolmogorov-Smirnov test. The Chi-square test was used to compare categorical variables. For within-group comparison a Wilcoxon matched-pair signed rank test was used. For comparison of between-groups differences Mann-Whitney test was applied and data are presented as mean ± SE of mean, unless otherwise stated. The mean value of the second SD was taken as the lower cut-off for non-measurable nerve conduction velocity when DSPN was clinically present. Because of the exploratory nature of the analyses, p-value < 0.05 was considered statistically significant and was not adjusted for multiplicity. Statistical data analysis was performed using GraphPad Prism 9 (https://www.graphpad.com/scientific-software/prism/, GraphPad Software, USA).

## 3 Results

### 3.1 Study design and participants

From the 41 originally randomized participants to M-Diet group (n = 19) and FMD group (n = 21), 31 (M-Diet group n = 14; FMD group n = 17) agreed to undergo extensive neuropathy assessment before and after diet intervention. One participant in the M-Diet group had a pacemaker and therefore was excluded from nerve conduction velocity study. 13 participants (M-Diet group n = 6; FMD group n = 7) consented to an MRN- imaging of the lower limb at baseline and at the end of study. Metabolic and anthropometric measurements at baseline were comparable between the two study groups. At note there were no differences in age, sex, BMI, glycemic, blood pressure and lipid control. All study participants were non-smokers (**Table 1**).

**Table 1:** Patient characteristics at baseline.

	M-Diet (n=14)	FMD (n=17)	p
**Age** (years)	67.1 ± 5.9	66.6 ± 5.8	0.81
**Sex** (females/males)	5/9	5/12	0.71
**BMI** (kg/m²)	30.2 ± 4.6	30.1 ± 4.1	0.95
**Diabetes duration** (years)	13.7 ± 8.0	14.5 ± 8.6	0.79
Comorbidities			
Hypertension n (%)	13 (93)	16 (94)	0.87
Coronary disease n (%)	6 (43)	5 (29)	0.53
Medication			
Oral antidiabetics n (%)	11 (79)	15 (88)	0.47
Insulin therapy n (%)	7 (50)	5 (29)	0.24
RAAS inhibitors n (%)	10 (71)	15 (88)	0.24
Beta Blockers n (%)	8 (57)	8 (53)	0.58
Statins n (%)	10 (71)	9 (53)	0.29
ASS n (%)	8 (57)	5 (29)	0.12
Glycemic control			
HbA1c (%)	7.8 ± 1.2	8.0 ± 1.5	0.69
FPG (mg/dl)	167.3 ± 49.0	164.8 ± 43.4	0.88
Blood pressure			
Systolic (mmHg)	143 ± 13	142 ± 14	0.84
Diastolic (mmHg)	83 ± 8	86 ± 9	0.34
Renal function			
Serum creatinine (mg/dl)	0.82 ± 0.29	0.88 ± 0.29	0.57
eGFR CKD-EPI (ml/min*1.73m²)	87.83 ± 17.62	83.86 ± 20.19	0.57
Serum lipids			
Triglycerides (mg/dl)	171 (63)	161 (135)	0.30
HDL (mg/dl)	46 ± 11	47 ± 13	0.82
LDL (mg/dl)	98 ± 35	88 ± 34	0.43
Clinical neuropathy assessment			
NSS/10	5.5 ± 3.4	5.4 ± 3.3	0.88
NDS/10	4.6 ± 3.1	3.5 ± 2.8	0.31
DSPN n (%)	9 (64)	8 (47)	0.34
Nerve conduction velocity			
Sural nerve sensory NCV (m/s)	38.54 ± 12.15	38.18 ± 7.45	0.77
Sural nerve SNAP (μV)	2.46 ± 2.29	3.96 ± 4.35	0.34
Tibial nerve motor NCV (m/s)	37.23 ± 8.58	39.29 ± 6.40	0.72
Tibial nerve CMAP (µV)	6.35 ± 5.31	7.79 ± 5.12	0.30
Peroneal nerve motor NCV (m/s)	37.39 ± 8.20	38.65 ± 7.65	0.76
Peroneal nerve CMAP (µV)	3.41 ± 2.83	5.05 ± 4.00	0.18
Quantitative sensory testing (z-scores)			
CDT	-1.73 ± 1.31	-1.13 ± 1.12	0.26
WDT	-1.37 ± 0.77	-0.77 ± 0.91	0.05
TSL	-1.57 ± 0.96	-1.06 ± 0.79	0.20
CPT	-0.42 ± 0.85	-0.45 ± 0.83	0.95
HPT	-0.76 ± 1.40	-0.02 ± 1.61	0.13
PPT	-0.38 ± 1.29	0.10 ± 1.22	0.46
MPT	0.71 ± 2.46	1.81 ± 2.00	0.26
MPS	0.18 ± 1.78	0.65 ± 1.24	0.46
WUR	-0.16 ± 0.80	-0.17 ± 0.94	0.70
MDT	-1.30 ± 2.40	-0.30 ± 2.81	0.90
VDT	-3.80 ± 2.96	-1.70 ± 2.67	**0.04**
Magnetic Resonance Neurography			
FA	0.37 ± 0.10	0.37 ± 0.05	0.76
T2-time (ms)	76.94 ± 17.57	72.85 ± 9.28	0.95

Data are shown as mean ± standard deviation (SD) for normally distributed variables, median (interquartile range) for log-normally distributed variables, or frequencies n (%) for categorical variables. P-values represent the difference between the study groups. Bold values represent statistical significance changes (P<0.05).

Parameters of quantitative sensory testing were compared to reference values, which were used to calculate z-scores for all single tests, except PHS and DMA: z-score positive values = hyperalgesia, z-score negative values = hypoesthesia or hypoalgesia.

M-Diet, Mediterranean diet; FMD, fasting-mimicking diet; BMI, body mass index; FPG, fasting plasma glucose; eGFR, estimated glomerular filtration rate; CKD-EPI, Chronic Kidney Disease Epidemiology Collaboration; HDL, high-density-lipoprotein; LDL, low-density-lipoprotein. NSS neuropathy symptom score; NDS, neuropathy disability score; DSPN, diabetic sensorimotor polyneuropathy; NCV nerve conduction velocity; SNAP, sensory nerve action potential; CMAP, compound motor action potential; CDT, cold detection threshold; WDT, warm detection threshold; TSL, thermal sensory limen; CPT, cold pain threshold; HPT, heat pain threshold; PPT, pain pressure threshold; MPT, mechanical pain threshold; MPS, mechanical pain sensitivity; WUR, wind-up ratio; MDT, mechanical detection threshold; VDT, vibration detection threshold; FA, sciatic nerve’s fractional anisotropy.

### 3.2 Neuropathy assessment

#### 3.2.1 Clinical neuropathy scores

At baseline, according to NDS and NSS scores, 9 (64%) participants of the M-Diet group and 8 (47%) participants of the FMD group had DSPN (**Table 1**) with 2 (14%) participants of the M-Diet and 0 participants of the FMD group reporting pain. 4 participants in of the M-Diet group (29%) and 1 participant of the FMD group (6%) had moderate DSPN (NDS and NSS≥ 6). Baseline values of clinical neuropathy scores were comparable with values after six-month diet intervention between study groups (NDS in the M-Diet group 4.6 ± 0.8 vs. 5.6 ± 1.0; P=0.34 and NDS in the FMD group 3.5 ± 0.7 vs. 3.4 ± 0.7; P=0.81, NSS in the M-Diet group 5.5 ± 0.9 vs. 5.6 ± 0.9; P=0.92 and NSS in the FMD group 5.4 ± 0.8 vs. 4.1 ± 1.0; P=0.21) (**Table 2**).

**Table 2:** Neuropathy assessment and metabolic outcomes.

Parameter		M-Diet	FMD	P
NDS	Baseline	4.6 ± 0.8	3.5 ± 0.7	
	After Intervention	5.6 ± 1.0	3.4 ± 0.7	0.27
NSS	Baseline	5.5 ± 0.9	5.4 ± 0.8	
	After Intervention	5.6 ± 0.9	4.1 ± 1.0	0.44
Sural nerve sensory NCV (m/s)	Baseline	38.54 ± 3.37	38.18 ± 1.81	
	After Intervention	38.54 ± 2.46	37.12 ± 2.04	0.82
Sural nerve SNAP (µV)	Baseline	2.46 ± 0.64	3.96 ± 1.06	
	After Intervention	2.10 ± 0.44	2.88 ± 0.69	0.63
Tibial nerve motor NCV (m/s)	Baseline	**37.23 ± 2.38**	39.29 ± 1.55	
	After Intervention	32.89 ± 3.05*	36.92 ± 2.38	0.45
Tibial nerve CMAP (µV)	Baseline	6.35 ± 1.47	**7.79 ± 1.24**	
	After Intervention	6.38 ± 1.49	9.21 ± 1.45*	0.13
Peroneal nerve motor NCV (m/s)	Baseline	37.39 ± 2.28	38.65 ± 1.86	
	After Intervention	37.08 ± 1.94	37.71 ± 1.94	>0.99
Peroneal nerve CMAP (µV)	Baseline	3.41 ± 0.79	5.05 ± 0.97	
	After Intervention	3.61 ± 0.72	4.77 ± 1.09	0.06
CDT	Baseline	-1.73 ± 0,35	-1.13 ± 0.28	
	After Intervention	-1.96 ± 0.37	-1.59 ± 0.25	0.79
WDT	Baseline	-1.37 ± 0.21	-0.77 ± 0,23	
	After Intervention	-1.46 ± 0.30	-0.95 ± 0.24	0.40
TSL	Baseline	-1.57 ± 0.26	-1,06 ± 0,20	
	After Intervention	-1.21 ± 0.30	-1.00 ± 0.19	0.19
CPT	Baseline	-0.42 ± 0.23	-0.45 ± 0,21	
	After Intervention	-0.46 ± 0.22	-0.28 ± 0.20	0.74
HPT	Baseline	**-0.76 ± 0.37**	-0.02 ± 0.36	
	After Intervention	-1.10 ± 0.30*	-0.47 ± 0.41	0.55
PPT	Baseline	-0.38 ± 0.36	0.10 ± 0,29	
	After Intervention	-0.47 ± 0.41	-0.09 ± 0.22	0.76
MPT	Baseline	0.71 ± 0.66	1.81 ± 0,48	
	After Intervention	0.36 ± 0.60	2.01 ± 0.55	0.40
MPS	Baseline	0.18 ± 0.49	0.65 ± 0,30	
	After Intervention	0.46 ± 0.52	0.77 ± 0.35	0.57
WUR	Baseline	-0.16 ± 0.24	-0.17 ± 0.23	
	After Intervention	0.59 ± 0.53	0.04 ± 0.23	0.76
MDT	Baseline	-1.30 ± 0.64	-0.30 ± 0.68	
	After Intervention	-1.52 ± 0.52	-0.78 ± 0.41	0.80
VDT	Baseline	-3.80 ± 0.79	-1.70 ± 0.65	
	After Intervention	-1.91 ± 0.89	-1.79 ± 0.62	0.34
FA	Baseline	0.37 ± 0.05	0.37 ± 0.02	
	After Intervention	0.37 ± 0.04	0.40 ± 0.02	0.64
T2-time	Baseline	76.94 ± 7.17	72.85 ± 3.51	
	After Intervention	75.21 ± 4.81	67.88 ± 3.35	0.37
FPG (mg/dL)	Baseline	167.3 ± 13.1	164.8 ± 10.5	
	After Intervention	153.0 ± 13.8	137.0 ± 12.7*	0.43
HbA1c (%)	Baseline	7.8 ± 0.3	**8.0 ± 0.4**	
	After Intervention	7.9 ± 0.3	6.7 ± 0.3**	**0.007**
Body weight (kg)	Baseline	93.8 ± 4.6	**92.9 ± 3.5**	
	After Intervention	92.7 ± 4.8	85.8 ± 3.6***	**0.0002**
BMI (kg/m^2^)	Baseline	30.2 ± 1.2	**30.1 ± 1.0**	
	After Intervention	29.9 ± 1.3	27.7 ± 1.0***	**0.06**
LDL (mg/dL)	Baseline	98.1 ± 9.8	88.0 ± 8.6	
	After Intervention	98.0 ± 9.9	88.9 ± 10.1	0.55
HDL (mg/dL)	Baseline	45.5 ± 2.9	46.5 ± 3.2	
	After Intervention	47.3 ± 2.9	48.8 ± 3.3	0.59
Triglycerides (mg/dL)	Baseline	187.1 ± 17.3	296.3 ± 92.6	
	After Intervention	188.9 ± 24.3	121.2 ± 12.4*	0.08
Blood ketones (mmol/L)	Baseline	0.12 ± 0.01	0.19 ± 0.06	
	After Intervention	0.11 ± 0.02	0.50 ± 0.12	**0.003**

Data are shown as mean ± SEM. P-value represents comparison of change between the groups. Bold values represent statistical significance changes (P<0.05).

Parameters of quantitative sensory testing were compared to reference values, which were used to calculate z-scores for all single tests, except PHS and DMA: z-score positive values = hyperalgesia, z-score negative values = hypoesthesia or hypoalgesia.

*P<0.05, **P<0.01, ***P<0.001 and represents the difference between baseline and after intervention values within the study group.

M-Diet, Mediterranean diet; FMD, fasting-mimicking diet; NSS, neuropathy symptom score; NDS, neuropathy disability score; NCV, nerve conduction velocity; SNAP, sensory nerve action potential; CMAP, compound motor action potential; CDT, cold detection threshold; WDT, warm detection threshold; TSL, thermal sensory limen; CPT, cold pain threshold; HPT, heat pain threshold; PPT, pain pressure threshold; MPT, mechanical pain threshold; MPS, mechanical pain sensitivity; WUR, wind-up ratio; MDT, mechanical detection threshold; VDT, vibration detection threshold; FA, sciatic nerve’s fractional anisotropy; FPG, fasting plasma glucose;, BMI, body mass index; FPG, fasting plasma glucose; LDL, low-density-lipoprotein; HDL, high-density-lipoprotein.

#### 3.2.2 Electrophysiological assessment

In the electrophysiological examinations there was no change observed in sensory nerve conduction velocity (NCV) of sural nerve in the M-Diet group (NCV 38.54 ± 3.37 m/s at baseline vs. 38.5 ± 2.46 m/s after intervention; P>0.9) and as well as no change was observed in the FMD group (NCV 38.18 ± 1.81 m/s at baseline vs. 37.12 ± 2.04 m/s after intervention; P=0.68). The differences in sensory NCV and SNAP of sural nerve after diet intervention were comparable between study groups (P=0.82 and P=0.63 respectively) (**Table 2**). Motor NCV of tibial nerve in the M-Diet group decreased after six months (motor NCV 37.23 ± 2.38 m/s at baseline vs. 32.89 ± 3.05 m/s after intervention; P=0.04), whereas this was not the case in the FMD group (motor NCV 39.29 ± 1.55 m/s at baseline vs. 36.92 ± 2.38 m/s after intervention; P=0.39). However, the difference in motor NCV of tibial nerve after diet intervention did not change between study groups (P=0.45) (**Table 2**). Tibial nerve CMAP did not change in M-Diet group (6.35 ± 1.47 µV at baseline vs. 6.38 ± 1.49 µV after intervention; P=0.81); and increased in the FMD group (7.79 ± 1.24 µV at baseline vs. 9.21 ± 1.45 µV after intervention; P=0.02). The difference in CMAP of tibial nerve after intervention did not change between study groups (P=0.13) (**Table 2**). Motor NCV of peroneal nerve remained unchanged in both study groups (M-Diet group 37.39 ± 2.28 m/s at baseline vs. 37.08 ± 1.94 m/s; P=0.20; and FMD group 38.65 ± 1.86 m/s at baseline vs. 37.71 ± 1.94 m/s after intervention; P=0.33) with no change between groups comparison (P>0.99). This was also the case of the CMAP of peroneal nerve (M-Diet group 3.41 ± 0.79 µV at baseline vs. 3.61 ± 0.72 µV after intervention; P=0.31; and FMD group 5.05 ± 0,97 µV at baseline vs. 4.77 ± 1.09 µV after intervention; P=0.26) with no change between groups comparison (P=0.06) (**Table 2**).

#### 3.2.3 Quantitative sensory testing

**Figure 1:**
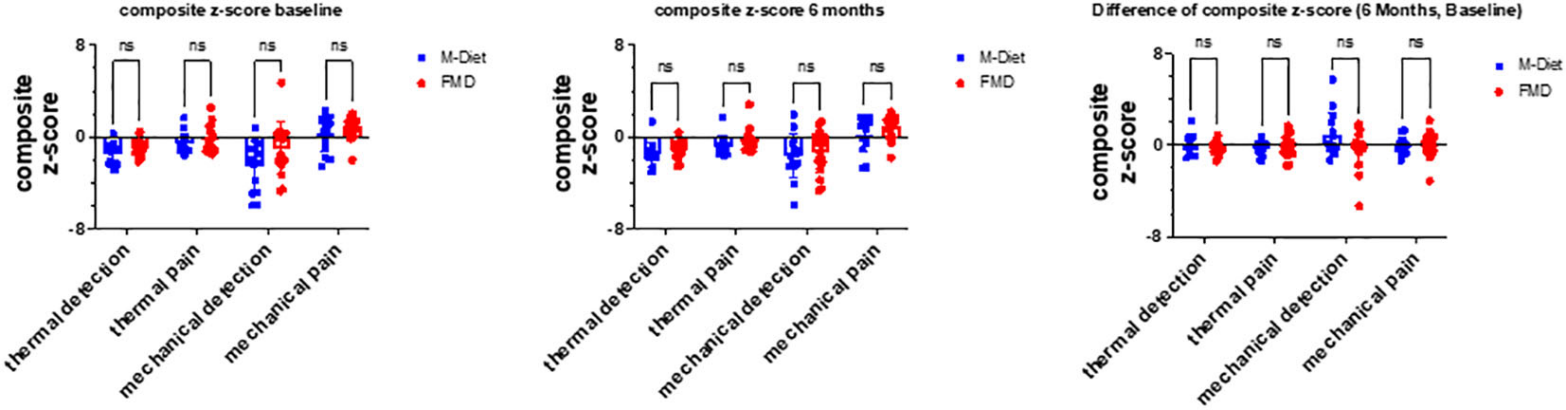
Neuropathy Assessment Composite z-Scores. Quantitative sensory testing (QST) parameters are plotted as composite z-scores: z-values of the single tests were clustered to: thermal detection (average values of CDT, WDT, and TSL, demonstrating thermoreceptive Aδ- and C-fiber function), thermal pain (average values of CPT and HPT, representing nociceptive C-fiber function), mechanical detection (average values of VDT and MDT, demonstrating tactile Aβ fiber function), and mechanical pain (average values of MPT, MPS, and PPT, representing nociceptive Aδ and C-fiber function). M-Diet, Mediterranean diet; FMD, fasting-mimicking diet, CDT, cold detection threshold; WDT, warm detection threshold; TSL, thermal sensory limen; CPT, cold pain threshold; HPT, heat pain threshold; PPT, pain pressure threshold; MPT, mechanical pain threshold; MPS, mechanical pain sensitivity; WUR, wind-up ratio; MDT, mechanical detection threshold; VDT, vibration detection threshold. ns, non-significant.

In quantitative sensory testing the control group showed a decrease by 45% in heat pain threshold, as a parameter assessing thermal pain (Z-score -0.76 ± 0.37 at baseline vs. -1.10 ± 0.30 after intervention; P=0.02), whereas no such change was observed in the FMD group (Z-score -0.02 ± 0.36 at baseline vs. -0.47 ± 0.41 after intervention; P=0.50). However, the difference did not change between study groups (P=0.55) (**Table 2**). Values of thermal detection, mechanical detection and mechanical pain after six months were comparable with baseline values within each study group and the differences to baseline did not differ between study groups (**Figure 1**).

#### 3.2.4 Metabolic control

One participant of the FMD group and one of the control group had not-measurable low-density-lipoprotein levels because of high triglyceride levels (>300 mg/dL) at baseline, and therefore were excluded from the analysis. Baseline anthropometric and metabolic measures were comparable between both groups (**Table 1**). The FMD group showed a reduction of HbA1c after intervention (8.0 ± 0.4% vs. 6.7 ± 0.3%; p <0.001), of body weight (92.9 ± 3.5 kg vs. 85.8 ± 3.6 kg; p<0.001) and of body mass index (BMI) (30.1 ± 1.0 kg/m² vs. 27.7 ± 1.0 kg/m²; p<0.001), as well as an increase in the blood ketones (0.19 ± 0.06 mmol/L vs. 0.50 ± 0.12 mmol/L) which were not observed in the M-Diet group (**Table 2**). Other metabolic parameters, such as fasting glucose levels and lipid profile did not change in both groups (**Table 2**). When adjusting the analysis of the neuropathy outcomes for weight loss and glycemic control improvement as we have previously reported (19) there is no change in the neuropathy parameters described above.

#### 3.2.5 Magnetic resonance neurography

**Figure 2:**
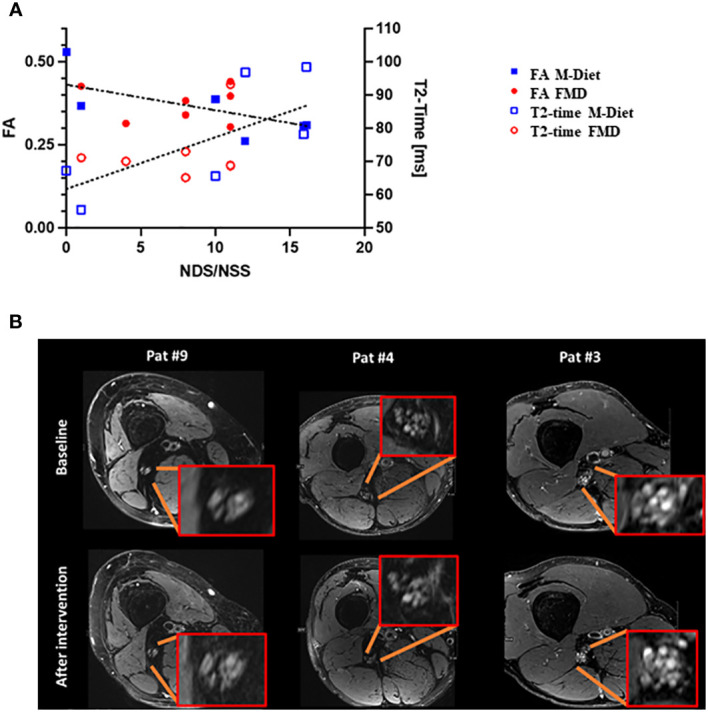
MRI and Neuropathy Assessment. **(A)**. Correlation of clinical degree of DSPN with quantitative MRN. Left y-axis indicating fractional anisotropy (FA) while right y-axis representing T2-time in milliseconds (ms). The x-axis represents the cumulated NDS/NSS score. Blue squares indicate patients of the M-diet group while red circles correspond to patients of the FMD group. A high degree of correlation was found between clinical degree of DSPN and both FA and T2-time as indicated by the linear fits (dashed and dotted lines). **(B)**. Representative images from both groups with matched imaging findings on high-resolution anatomical T2-weighted MRN over time. Patient 4 and 9 were in the M-Diet group, and patient 3 was in the FMD group. Nerve structure including hyperintense fascicular lesions appear highly stable over the six-month intervention period irrespective of treatment group or the degree of structural pathology upon initial presentation. M-Diet, Mediterranean diet; FMD, fasting-mimicking diet; FA, sciatic nerve’s fractional anisotropy; NSS, neuropathy symptom score; NDS, neuropathy disability score. ns, non-significant.

Consistent with previous results, neuropathy scores correlated negatively with FA (R²=0.34, p=0.04) and positively with T2-time (R²=0.4, p=0.02) (**Figure 2A**).

High-resolution T2-weighted anatomical MRN imaging showed consistent hyperintense fascicular nerve lesions in both groups over time – with no evidence of relevant changes during the intervention and irrespective of the degree of initial findings (**Figure 2B**). This qualitative observation was further supported when comparing the study groups separately with regard to the quantitative MRN markers FA and T2-time: neither the FMD nor the M-Diet group showed changes: FA values (p=0.16 for the FMD group and p=0.81 for the M-Diet) and T2-time values (p=0.3 for the FMD-group and p>0.99 for the M-Diet) (**Table 2**).

## 4 Discussion

This study explored in a randomized controlled setting the clinical effects of periodic fasting on somatosensory nerve function in individuals with T2D. We found at the end of study no changes in clinical neuropathy scores within the study groups, especially no worsening of somatosensory nerve function after improvement of glycemic control under periodic fasting. Moreover, we found a decrease in motor NCV of N. tibialis in the M-Diet group and an increase in N. tibialis CMAP in the FMD group. The participants of the M-Diet group had a decrease in heat pain threshold. MRN analysis confirmed the correlation between fascicular nerve lesions of the sciatic nerve and clinical neuropathy scores and no changes between the groups were observed after intervention.

Pathophysiology of DSPN remains still debatable and multiple factors can contribute to its clinical presentation of most commonly “stocking and glove” pattern (29). Although DSPN is not considered primarily a demyelinating neuropathy, Schwann cells are targeted by chronic hyperglycemia, and severe cases of DSPN include features of demyelination (30, 31). Weekly administration of FMD once a month over a period of six months has already been shown to significantly reduce albuminuria and improve glycemic control in individuals with T2D and diabetic nephropathy, showing a comparable effect size as reported from interventions with SGLT2-inhibitors (19, 32). We therefore investigated treatment effects of FMD on DSPN, as another microvascular complication of diabetes. FMD has shown to ameliorate symptoms in a murine experimental autoimmune encephalomyelitis model via promoting oligodendrocyte precursor-dependent regeneration and reducing the levels of immune-cells including T cells, monocytes, and central nervous system specific immune-cells as microglia (33). These effects could be possibly mediated by endogenous glucocorticoid production induced by FMD (33).

Studies investigating the effect of lifestyle intervention either with a diet or a physical intervention in DSPN that have been previously reported, are done in few controlled studies and not widely agreeing on the best readouts (34). Long-term intensive lifestyle intervention with study duration 9-11 years leads to a decrease in questionnaire-based DSPN dependent on weight loss, changes in HbA1c and lipid levels (11). In this respect, we could show that six-month periodic fasting decreases body weight and improves glycemic control in individuals with T2D, becoming in this way a potential therapeutic tool also for the treatment of DSPN (19). Although the underlying mechanism of TIND remain unknown (18) rapid changes in glycemic control seem to relate with the parallel development of TIND and other microvascular complication such as diabetic retinopathy (35). In this study we find no detrimental effects of periodic fasting on sensorimotor nerve function, despite the improvement in glycemic control, without initiating treatment-induced diabetic neuropathy (19). Aerobic exercise has achieved symptom-reduction of painful neuropathy (36). Accordingly, a randomized 4 year-long supervised aerobic activity study has shown improvement in nerve conductive velocity with potential to modify onset of DSPN (10).

Of note, our study compared the effects of weekly administration of FMD with those of a Mediterranean diet. In this way the diet protocol could be standardized to some extent also for the control group, since the diet protocol was tightly regulated for the intervention group. This approach avoided substantial differences in food intake between the participants of the control group, maintaining nutritional homogeneity within the group. Mediterranean diet has been shown to be beneficial for glycemic control in type 2 diabetes patients (37) and by offering regular dietary consultation to the participants of the control group, we could achieve same contact time between study participant and study medical team.

Despite a significant weight loss after intervention in the FMD group, when adjusting the statistical analysis for weight loss we found no changes in the reported neuropathy outcomes, suggesting that weight loss alone is not sufficient to modify neuropathy outcomes in this study setting. Positive associations between chronic caloric restriction with weight-loss and thermal nociception have been previously described in animal models with mice exhibiting higher hot-plate latencies after tail-amputation on caloric restriction (40% reduction of caloric intake over 100 weeks) in comparison to ad libitum fed controls. Possible mechanisms involved changes of analgesia-regulating hormones such as glucocorticoids, opioid receptors and adrenocortical hormones modified by short-term food deprivation (38). An intermittent fasting diet model in mice with stable weight has also induced reduction in nociceptive response by provoking upregulation of opiate receptor expression in spinal cord (17). Comparison of different pain models has shown different magnitude on pain modulation by fasting suggesting a stronger effect on nociception than visceral pain. Of note, lower phosphorylation levels of ERK and mTOR in the dorsal root ganglia and spinal cord could be measured in the fasting group in comparison to control, suggesting that inhibition of pathomechanisms that lead to insulin resistance might be responsible for the antinociceptive effects of fasting (16). Indeed, the participants of the FMD group had an improvement of insulin resistance marker, glycemia and weight loss as we recently reported (19).

The rising incidence of DSPN with longer disease duration of diabetes is naturally associated with ageing process. Caloric restriction as a therapeutic approach to neuropathic pain induced by peripheral nerve damage in elderly mice (12 months old) has been shown to increase mechanical threshold reflecting clinical improvement (39). In our human study we could show that periodic fasting leads to a reduction of senescence marker, suggesting that periodic fasting might protect against age-related diseases (19). Deficiency in cell autophagy can exacerbate allodynia which can be partially counteracted by caloric restriction in mice (40).

Although previous studies have demonstrated excellent interobserver agreement for neuropathy assessment (41), observer bias remains still debatable. Dyck et al. found a poor interobserver reproducibility amongst expert neurophysiologist who independently assessed nerve conduction parameters in the same patients with diabetes (42). In our study, all NCS were done by the same well-trained medical technician, avoiding in this way interobserver reproducibility concerns. Moreover, most clinical trials on diabetic neuropathy have used NCS as the primary outcome to assess treatment efficacy (43–45). A multicenter study has demonstrated that standardized QST performed by trained examiners is a valuable diagnostic instrument with good test-retest and interobserver reproducibility and with standardized training, observer bias is much lower than random variance (46). The QST measurements were performed based on a standardized QST protocol of the German Research Network on Neuropathic Pain (DFNS) by 2 well-trained medical technicians (24). All investigators performing QST were trained and certified by the Department of Neurophysiology at the University Hospital of Mannheim.

MRN can visualize and characterize peripheral nerve structures including internal fascicular patterns. MRN analysis was offered to all study participants, however as an optional test. The major reasons for not conducting an MRN analysis were non-consenting (n=7 in the control group and n=9 in the FMD group) and restrictions due to Covid-19 pandemic (n=1 in the control group and n=1 in the FMD group). Although we could not perform MRN in all study participants, we could still confirm the correlation between fascicular nerve lesions of the sciatic nerve and clinical neuropathy scores as previously reported (47–50). MRN changes were found to be stable over time irrespective of their initial extent and quantitative nerve integrity MRN parameters did not change between the groups after the six-month intervention time. This argues for a rather slow pathophysiological process for these structural neve changes to occur. On the other hand, MRN findings need to be carefully interpreted and further follow-up studies including larger sample sizes over a longer observational period are needed.

Noteworthy our study has several limitations. The intervention duration of six months is relatively short for investigating the course of DSPN and limits possible effects of periodic fasting on delaying progression rather than reversing positive symptoms or improving functionality. Previous studies have shown increase of cutaneous nerve density after one year of diet intervention in patients with diabetes mellitus (51) as well as in the state of impaired glucose tolerance (28). However, our study offers a very good controlled diet intervention and as a consequence a high compliance rate. Despite the short-term diet intervention, our study offers an extensive assessment of sensory somatosensory nerve function including, including electrophysiological, QST and MRN analysis. Our study cohort was small and the M-Diet group had a stronger deteriorated nerve function than the FMD group at baseline. This might explain the worsening of DSPN in the M-Diet group observed at the end of the study, reflected by worsening of Tibial nerve motor NCV and HPT (**Table 2**), with therefore changes towards a progression of DSPN after study intervention. However, baseline metabolic and anthropometric characteristics, including baseline characteristics of DSPN, did not differ significantly between the study groups and the differences observed after intervention did not change for all analyzed parameters. Our study group had a mild phenotype of DSPN according to the clinical neuropathy scores and to the QST z-score values within the second standard deviation, challenging on one hand clinically relevant changes in the course of DSPN in this intervention, and making it on the other hand more prone to be affected by a diet intervention. In a *post-hoc* analysis for estimating the effect size based on the NDS and NSS score observed in this study we found a Cohen’s d effect size of 0.4. Therefore, the achieved effect size in this study is smaller than the hypothesized effect size (1.1). These results point out the challenges for designing future studies with larger cohorts and stronger interventions that might offer clinical relevance and generalizability in patients with type 2 diabetes and DSPN.Considering the explorative nature of this study we did not adjust for multiple comparison analysis. However, we are aware of the risk for spurious findings and future mechanistic studies are needed to investigate the effects of periodic fasting on nerve function.

As a conclusion our study shows that six-month periodic fasting was safe and had no deleterious effects on the clinical course of DSPN in type 2 diabetes patients. Taken together, fasting and diet intervention remain intriguing therapeutic approaches for DSPN with a still unfulfilled need on investigating fasting effects in big cohort studies, with long study intervention and in severe forms of DSPN.

## Data availability statement

The raw data supporting the conclusions of this article will be made available from the corresponding author upon reasonable request for research purpose, after anonymization and approval by the local ethics committee. The datasets presented in this article are subject to national data protection laws and restrictions imposed by the ethics committee to ensure the privacy of study participants.

## Ethics statement

The studies involving human participants were reviewed and approved by Ethics Committee Medical Faculty Heidelberg. The patients/participants provided their written informed consent to participate in this study.

## Author contributions

AS contributed to conception of work and study design. ZK, ER and AS contributed to the acquisition and interpretation of data. DT, LS, HB, SK, VL and StH contributed to the interpretation of data. ZK, ER, JS and AS drafted the report. AS and JS are the guarantors of this work and, as such, had full access to all the data in the study and take responsibility for the integrity of the data and the accuracy of the data analysis. All authors contributed to the article and approved the submitted version. 

## References

[1] IDF diabetes atlas 2021 | IDF diabetes atlas. Available at: https://diabetesatlas.org/atlas/tenth-edition/. 10.1016/j.diabres.2015.05.037 26119773

[2] Global report on diabetes. Available at: https://www.who.int/publications-detail-redirect/9789241565257.

[3] Vinik AI Nevoret ML Casellini C Parson H. Diabetic neuropathy. Endocrinol Metab Clin North Am (2013) 42(4):747–87. doi: 10.1016/j.ecl.2013.06.001 24286949

[4] Partanen J Niskanen L Lehtinen J Mervaala E Siitonen O Uusitupa M. Natural history of peripheral neuropathy in patients with non-Insulin-Dependent diabetes mellitus. N Engl J Med (1995) 333(2):89–94. doi: 10.1056/NEJM199507133330203 7777034

[5] Yu Y. Gold standard for diagnosis of DPN. Front Endocrinol (2021) 12:719356. doi: 10.3389/fendo.2021.719356 PMC8576350 34764937

[6] Feldman EL Callaghan BC Pop-Busui R Zochodne DW Wright DE Bennett DL. Diabetic neuropathy. Nat Rev Dis Primer. (2019) 5(1):41. doi: 10.1038/s41572-019-0092-1 31197153

[7] Cernea S Raz I. Management of diabetic neuropathy. Metabolism (2021) 123:154867. doi: 10.1016/j.metabol.2021.154867 34411554

[8] Azmi S Alam U Burgess J Malik RA. State-of-the-art pharmacotherapy for diabetic neuropathy. Expert Opin Pharmacother. (2021) 22(1):55–68. doi: 10.1080/14656566.2020.1812578 32866410

[9] Callaghan BC Little AA Feldman EL Hughes RA. Enhanced glucose control for preventing and treating diabetic neuropathy. Cochrane Database Syst Rev. (2012) 6(6):CD007543. doi: 10.1002/14651858.cd007543.pub2 PMC4048127 22696371

[10] Balducci S Iacobellis G Parisi L Di Biase N Calandriello E Leonetti F. Exercise training can modify the natural history of diabetic peripheral neuropathy. J Diabetes Complications. (2006) 20(4):216–23. doi: 10.1016/j.jdiacomp.2005.07.005 16798472

[11] Look AHEAD Research Group. Effects of a long-term lifestyle modification programme on peripheral neuropathy in overweight or obese adults with type 2 diabetes: the look AHEAD study. Diabetologia (2017) 60(6):980–8. doi: 10.1007/s00125-017-4253-z PMC5423967 28349174

[12] Ułamek-Kozioł M Czuczwar SJ Januszewski S Pluta R. Ketogenic diet and epilepsy. Nutrients (2019) 11(10):2510. doi: 10.3390/nu11102510 31635247 PMC6836058

[13] Storoni M Plant GT. The therapeutic potential of the ketogenic diet in treating progressive multiple sclerosis. Mult Scler Int (2015) 2015:681289. doi: 10.1155/2015/681289 26839705 PMC4709725

[14] Yarar-Fisher C Kulkarni A Li J Farley P Renfro C Aslam H. Evaluation of a ketogenic diet for improvement of neurological recovery in individuals with acute spinal cord injury: a pilot, randomized safety and feasibility trial. Spinal Cord Ser Cases. (2018) 4:88. doi: 10.1038/s41394-018-0121-4 PMC6155083 30275980

[15] Zeng H Lu Y Huang MJ Yang YY Xing HY Liu XX. Ketogenic diet-mediated steroid metabolism reprogramming improves the immune microenvironment and myelin growth in spinal cord injury rats according to gene and co-expression network analyses. Aging (2021) 13(9):12973–95. doi: 10.18632/aging.202969 PMC8148504 33962394

[16] Jang SP Park SH Jung JS Lee HJ Hong JW Lee JY. Characterization of changes of pain behavior and signal transduction system in food-deprived mice. Anim Cells Syst (2018) 22(4):227–33. doi: 10.1080/19768354.2018.1490348 PMC6138332 30460102

[17] de los Santos-Arteaga M Sierra-Domínguez SA Fontanella GH Delgado-García JM Carrión ÁM. Analgesia induced by dietary restriction is mediated by the κ-opioid system. J Neurosci (2003) 23(35):11120–6. doi: 10.1523/JNEUROSCI.23-35-11120.2003 PMC6741040 14657170

[18] Gibbons CH Freeman R. Treatment-induced neuropathy of diabetes: an acute, iatrogenic complication of diabetes. Brain (2015) 138(1):43–52. doi: 10.1093/brain/awu307 25392197 PMC4285188

[19] Sulaj A Kopf S von Rauchhaupt E Kliemank E Brune M Kender Z. Six-month periodic fasting in patients with type 2 diabetes and diabetic nephropathy: a proof-of-Concept study. J Clin Endocrinol Metab (2022) 107(8):2167–81. doi: 10.1210/clinem/dgac197 PMC9282263 35661214

[20] Arzneimittelkommission Der Deutschen Ärzteschaft (AkdÄ) Deutsche Diabetes-Gesellschaft (DDG) Deutsche Gesellschaft Für Allgemeinmedizin Und Familienmedizin (DEGAM) Deutsche Gesellschaft Für Anästhesiologie Und Intensivmedizin (DGAI) Deutsche Gesellschaft Für Innere Medizin (DGIM) Vertreten Durch Die (DDG). Nationale VersorgungsLeitlinie neuropathie bei diabetes im erwachsenenalter - langfassung. 1. Auflage. Version 5. Bundesärztekammer (BÄK), Kassenärztliche Bundesvereinigung (KBV), Arbeitsgemeinschaft der Wissenschaftlichen Medizinischen Fachgesellschaften (AWMF). (2011). doi: 10.6101/AZQ/000302.

[21] Carmichael J Fadavi H Ishibashi F Shore AC Tavakoli M. Advances in screening, early diagnosis and accurate staging of diabetic neuropathy. Front Endocrinol (2021) 12:671257. doi: 10.3389/fendo.2021.671257 PMC8188984 34122344

[22] Ziegler D Bönhof GJ Strom A Straßburger K Karusheva Y Szendroedi J. Progression and regression of nerve fibre pathology and dysfunction early in diabetes over 5 years. Brain (2021) 144(10):3251–63. doi: 10.1093/brain/awab330 34499110

[23] Tesfaye S Boulton AJM Dyck PJ Freeman R Horowitz M Kempler P. Diabetic neuropathies: update on definitions, diagnostic criteria, estimation of severity, and treatments. Diabetes Care (2010) 33(10):2285–93. doi: 10.2337/dc10-1303 PMC2945176 20876709

[24] Rolke R Baron R Maier C Tölle TR Treede--R Beyer A. Quantitative sensory testing in the German research network on neuropathic pain (DFNS): standardized protocol and reference values. PAIN (2006) 123(3):231–43. doi: 10.1016/j.pain.2006.01.041 16697110

[25] Kopf S Groener JB Kender Z Fleming T Bischoff S Jende J. Deep phenotyping neuropathy: an underestimated complication in patients with pre-diabetes and type 2 diabetes associated with albuminuria. Diabetes Res Clin Pract (2018) 146:191–201. doi: 10.1016/j.diabres.2018.10.020 30389624

[26] Groener JB Jende JME Kurz FT Kender Z Treede RD Schuh-Hofer S. Understanding diabetic neuropathy–from subclinical nerve lesions to severe nerve fiber deficits: a cross-sectional study in patients with type 2 diabetes and healthy control subjects. Diabetes (2020) 69(3):436–47. doi: 10.2337/db19-0197 31826867

[27] Schwarz D Hidmark AS Sturm V Fischer M Milford D Hausser I. Characterization of experimental diabetic neuropathy using multicontrast magnetic resonance neurography at ultra high field strength. Sci Rep (2020) 10:7593. doi: 10.1038/s41598-020-64585-1 32371885 PMC7200726

[28] Smith AG Russell J Feldman EL Goldstein J Peltier A Smith S. Lifestyle intervention for pre-diabetic neuropathy. Diabetes Care (2006) 29(6):1294–9. doi: 10.2337/dc06-0224 16732011

[29] Yang H Sloan G Ye Y Wang S Duan B Tesfaye S. New perspective in diabetic neuropathy: from the periphery to the brain, a call for early detection, and precision medicine. Front Endocrinol (2020) 10:929. doi: 10.3389/fendo.2019.00929 PMC6978915 32010062

[30] Dunnigan SK Ebadi H Breiner A Katzberg HD Lovblom LE Perkins BA. Conduction slowing in diabetic sensorimotor polyneuropathy. Diabetes Care (2013) 36(11):3684–90. doi: 10.2337/dc13-0746 PMC3816879 24026550

[31] Gumy LF Bampton ETW Tolkovsky AM. Hyperglycaemia inhibits schwann cell proliferation and migration and restricts regeneration of axons and schwann cells from adult murine DRG. Mol Cell Neurosci (2008) 37(2):298–311. doi: 10.1016/j.mcn.2007.10.004 18024075

[32] Cherney D Lund SS Perkins BA Groop PH Cooper ME Kaspers S. The effect of sodium glucose cotransporter 2 inhibition with empagliflozin on microalbuminuria and macroalbuminuria in patients with type 2 diabetes. Diabetologia (2016) 59(9):1860–70. doi: 10.1007/s00125-016-4008-2 27316632

[33] Choi IY Piccio L Childress P Bollman B Ghosh A Brandhorst S. A diet mimicking fasting promotes regeneration and reduces autoimmunity and multiple sclerosis symptoms. Cell Rep (2016) 15(10):2136–46. doi: 10.1016/j.celrep.2016.05.009 PMC4899145 27239035

[34] Zilliox LA Russell JW. Physical activity and dietary interventions in diabetic neuropathy: a systemic review. Clin Auton Res Off J Clin Auton Res Soc (2019) 29(4):443–55. doi: 10.1007/s10286-019-00607-x PMC6697618 31076938

[35] Gibbons CH Freeman R. Treatment-induced diabetic neuropathy: a reversible painful autonomic neuropathy. Ann Neurol (2010) 67(4):534–41. doi: 10.1002/ana.21952 PMC3057039 20437589

[36] Kluding PM Pasnoor M Singh R Jernigan S Farmer K Rucker J. The effect of exercise on neuropathic symptoms, nerve function, and cutaneous innervation in people with diabetic peripheral neuropathy. J Diabetes Complications. (2012) 26(5):424–9. doi: 10.1016/j.jdiacomp.2012.05.007 PMC3436981 22717465

[37] Schwingshackl L Chaimani A Hoffmann G Schwedhelm C Boeing H. A network meta-analysis on the comparative efficacy of different dietary approaches on glycaemic control in patients with type 2 diabetes mellitus. Eur J Epidemiol. (2018) 33(2):157–70. doi: 10.1007/s10654-017-0352-x PMC5871653 29302846

[38] Hargraves WA Hentall ID. Analgesic effects of dietary caloric restriction in adult mice. Pain (2005) 114(3):455–61. doi: 10.1016/j.pain.2005.01.010 15777870

[39] De Angelis F Vacca V Pavone F Marinelli S. Impact of caloric restriction on peripheral nerve injury-induced neuropathic pain during ageing in mice. Eur J Pain. (2020) 24(2):374–82. doi: 10.1002/ejp.1493 31610068

[40] Coccurello R Nazio F Rossi C De Angelis F Vacca V Giacovazzo G. Effects of caloric restriction on neuropathic pain, peripheral nerve degeneration and inflammation in normometabolic and autophagy defective prediabetic Ambra1 mice. PloS One (2018) 13(12):e0208596. doi: 10.1371/journal.pone.0208596 30532260 PMC6287902

[41] Litchy WJ Albers JW Wolfe J Bolton CF Walsh N Klein CJ. Proficiency of nerve conduction using standard methods and reference values (Cl. NPhys trial 4). Muscle Nerve. (2014) 50(6):900–8. doi: 10.1002/mus.24243 PMC4169346 24644133

[42] Dyck PJ Overland CJ Low PA Litchy WJ Davies JL Dyck PJB. Signs and symptoms versus nerve conduction studies to diagnose diabetic sensorimotor polyneuropathy: cl vs. NPhys trial. Muscle Nerve. (2010) 42(2):157–64. doi: 10.1002/mus.21661 PMC2956592 20658599

[43] Malik RA Williamson S Abbott C Carrington AL Iqbal J Schady W. Effect of angiotensin-converting-enzyme (ACE) inhibitor trandolapril on human diabetic neuropathy: randomised double-blind controlled trial. Lancet Lond Engl (1998) 352(9145):1978–81. doi: 10.1016/S0140-6736(98)02478-7 9872248

[44] Wahren J Foyt H Daniels M Arezzo JC. Long-acting c-peptide and neuropathy in type 1 diabetes: a 12-month clinical trial. Diabetes Care (2016) 39(4):596–602. doi: 10.2337/dc15-2068 26884473

[45] Kennedy WR Navarro X Goetz FC Sutherland DE Najarian JS. Effects of pancreatic transplantation on diabetic neuropathy. N Engl J Med (1990) 322(15):1031–7. doi: 10.1056/NEJM199004123221503 2320063

[46] Geber C Klein T Azad S Birklein F Gierthmühlen J Huge V. Test-retest and interobserver reliability of quantitative sensory testing according to the protocol of the German research network on neuropathic pain (DFNS): a multi-centre study. Pain (2011) 152(3):548–56. doi: 10.1016/j.pain.2010.11.013 21237569

[47] Jende JME Kender Z Mooshage C Groener JB Alvarez-Ramos L Kollmer J. Diffusion tensor imaging of the sciatic nerve as a surrogate marker for nerve functionality of the upper and lower limb in patients with diabetes and prediabetes. Front Neurosci (2021) 15:642589. doi: 10.3389/fnins.2021.642589 33746707 PMC7966816

[48] Bäumer P Weiler M Ruetters M Staub F Dombert T Heiland S. MR neurography in ulnar neuropathy as surrogate parameter for the presence of disseminated neuropathy. PloS One (2012) 7(11):e49742. doi: 10.1371/journal.pone.0049742 23166762 PMC3498206

[49] Pham M Oikonomou D Hornung B Weiler M Heiland S Bäumer P. Magnetic resonance neurography detects diabetic neuropathy early and with proximal predominance. Ann Neurol (2015) 78(6):939–48. doi: 10.1002/ana.24524 PMC5132066 26381658

[50] Jende JME Groener JB Oikonomou D Heiland S Kopf S Pham M. Diabetic neuropathy differs between type 1 and type 2 diabetes: insights from magnetic resonance neurography. Ann Neurol (2018) 83(3):588–98. doi: 10.1002/ana.25182 29443416

[51] Singleton JR Marcus RL Jackson JE K Lessard M Graham TE Smith AG. Exercise increases cutaneous nerve density in diabetic patients without neuropathy. Ann Clin Transl Neurol (2014) 1(10):844–9. doi: 10.1002/acn3.125 PMC4241811 25493275

